# Collaboration between a temperate phage and *Pseudomonas aeruginosa* quorum sensing constrains social cheats

**DOI:** 10.1128/mbio.02360-25

**Published:** 2025-09-22

**Authors:** Ruiyi Chen, Beth Traxler, Andrew M. Kropinski, E. Peter Greenberg

**Affiliations:** 1 Department of Microbiology, University of Washington School of Medicine 12353 , Seattle, Washington, USA; 2 Department of Pathobiology, Ontario Veterinary College, University of Guelph 3653, Guelph, Ontario, Canada; NYU Langone Health, New York, New York, USA

**Keywords:** Bacterial communication, cell-cell signaling, LasR, sociomicrobiology

## Abstract

*Pseudomonas aeruginosa* uses quorum sensing (QS) to coordinate cooperative behaviors. In the model strain PAO1, QS activates dozens of genes, including those for cyanide production and cyanide resistance. The induction of cyanide synthesis and resistance mechanisms serves to constrain the emergence of QS mutants. Strain CI27 does not possess the cyanide synthesis genes. Here, we ask if this isolate can constrain emergence of QS mutants. In daily transfer experiments, where PAO1 cyanide synthesis mutants are incapable of QS mutant restraint, we found that CI27 constrained emergence of QS mutants. Genome sequencing revealed that a temperate phage, which we call RC5, in evolved QS mutants had copied itself at new chromosomal locations. This led to investigations of the relationship between QS and temperate phages in CI27. We found seven prophage genomes in the CI27 chromosome (RC1–7), one of which occurred in two copies (RC3). Upon mitomycin C induction of CI27, we found four different phage genomes in lysates with RC7 predominating. The lysates of a QS mutant contained three times as many phages as lysates. Without mitomycin C induction, phage numbers in culture fluid from either the wild-type or a CI27 QS mutant were low, but upon daily transfer, phage numbers increased dramatically in the QS mutant. The increase was due to RC3 specifically, and after several transfers, the mutant cultures underwent a massive lysis. Further experiments indicated that RC3 protects *P. aeruginosa* from QS mutant invasion. Protection might result at least in part from the fact that LasR mutants are more susceptible to RC3 superinfection than the wild type.

**IMPORTANCE:**

Quorum sensing (QS) enables bacteria such as *Pseudomonas aeruginosa* to coordinate cooperative activities. How bacteria in cooperating groups can resist infiltration by non-cooperating variants is an emerging area of interest in sociobiology and molecular biology. There have been several recent reports on how QS and certain bacteriophage interact. In some strains of *P. aeruginosa*, QS can activate phage defense systems. At least one bacteriophage can repress *P. aeruginosa* QS. Here, we show that a previously undescribed bacteriophage can help cooperating groups of *P. aeruginosa* resist infiltration by non-cooperating QS mutants. This represents a mutualism in which both the bacteriophage and the *P. aeruginosa* host benefit at least under certain conditions.

## INTRODUCTION

In *Pseudomonas aeruginosa*, there are two LuxI-LuxR-type acylhomoserine lactone (AHL) quorum sensing (QS) systems, which are involved in cell-density-dependent activation of dozens to hundreds of genes depending on the isolate studied (1). One particular isolate, PAO1, has been studied intensively with respect to QS, and it has been used as a general model to understand QS in the context of coordinating cooperative activities. In PAO1, the LasIR system activates dozens of genes including those coding for the other circuit, RhlIR, and the *Pseudomonas* quinolone signal (PQS) circuit (2, 3). *P. aeruginosa* activates expression of genes encoding extracellular proteases by QS, and thus, an intact LasIR system is required for growth on proteins such as casein. When PAO1 is transferred daily in broth with casein as the sole source of carbon and energy, LasR mutants emerge and reach about 25–40% of the total population (4, 5). These LasR mutants meet the definition of social cheaters. They do not produce extracellular proteases, the public goods required for growth, but they invade the cooperating group by using the proteases produced by the wildtype (WT) members of the population. Through the activation *of rhlIR*, LasR activates cyanide production and also a cyanide resistance gene (6, 7). Cyanide synthesis mutants cannot control the emergence of LasR mutant cheaters, which are sensitive to cyanide, and the cheater frequency in evolved populations can be 80–90% of the total. When LasR mutants reach this level, the ability of the cooperators to sustain population growth is lost (6).

We previously examined the QS regulons of six *P. aeruginosa* isolates other than PAO1 (1). One of these isolates, CI27, showed a QS regulon, which overlapped extensively with that of PAO1, but this isolate had a genome deletion encompassing *hcnABC*, the operon required for cyanide synthesis. Here, we asked whether this isolate is capable of restraining emergence of LasR mutant cheaters when transferred daily on casein, and if so, how? This led to the discovery that CI27 is a polylysogen containing seven different prophage genomes. LasR mutants are particularly sensitive to one of these phages, even if lysogenized by it. In this way, the phage collaborates with QS cooperators to restrain emergence of LasR mutants.

## RESULTS

### Emergence of CI27 LasR social cheats is constrained by LasR-competent cooperators during growth on casein

**Fig 1:**
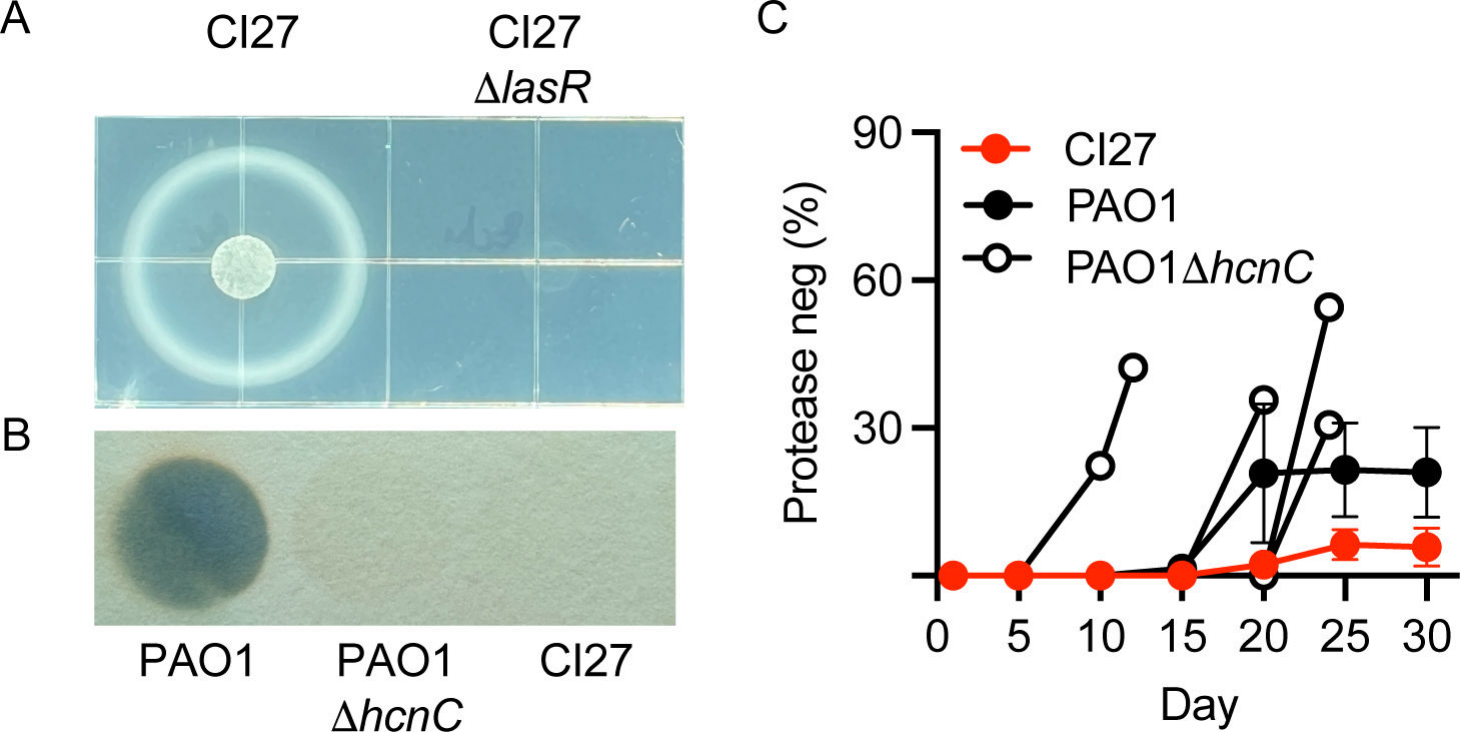
Strain CI27 grows on casein, does not produce cyanide, and constrains emergence of protease-negative mutants during passage on casein broth. (**A**) CI27 and a CI27 *lasR*-deletion mutant were spotted on casein agar. A colony of the WT surrounded by a halo of partially digested casein protein was evident after 24 h at 37°C, whereas only a faint spot of the *lasR* deletion mutant was evident. (**B**) Cyanide production monitored with a cyanogenic paper assay. PAO1 produced detectable cyanide but not the PAO1 *hcnC* deletion mutant or CI27 WT. (**C**) CI27, PAO1, and the PAO1 *hcnC* deletion mutant grown in casein broth were transferred daily. Screening for protease-negative cells was at five-day intervals. For PAO1 Δ*hcnC*, we did not observe growth after the last day, on which data are shown.

Our previous publication indicated that CI27 has functional QS circuitry, but carries a deletion encompassing the *hcnABC* operon (1). To begin, we confirmed that it exhibits LasR-dependent growth on casein and, as expected, does not produce cyanide like PAO1 does (Fig. 1A and B).

When WT PAO1 is grown on casein as the sole source of carbon and energy, it requires the secretion of QS-induced proteases to digest the protein into peptides and amino acids for uptake and further metabolism. When PAO1 is transferred on casein daily, LasR mutant cheats emerge and come to equilibrium with cooperators. The LasR mutants can be putatively identified as protease-negative colonies on skim milk agar (4). This equilibrium involves the activation of the genes for cyanide synthesis in the LasR WT (6). We asked whether CI27, which lacks the *hcn* gene cluster, has the ability to restrain LasR mutants or whether it behaves like a PAO1 cyanide-negative *hcnC* mutant. As controls for our casein passage experiment, we used WT PAO1 and a PAO1 *hcnC* deletion mutant. As expected, protease-negative PAO1 mutants were detected 20 days after daily transfers and reached about 20%–40% of the population. In four independent lineages of the PAO1 cyanide-negative mutant, protease-negative cheats were detected as early as day 10 and reached higher levels than those cheats observed in the WT lineages. Shortly after PAO1 protease-negative mutants were detected, the growth of the cyanide mutant was not sustained on transfer. With five independent CI27 lineages, protease-negative mutants were detected at day 20 or later and reached a maximum of <10% (Fig. 1C). These results are consistent with previous reports that WT *P. aeruginosa* can constrain emergence of QS mutants during growth on casein. In strain PAO1, this requires QS activation of *hcn* genes. In CI27, there must be some other constraining mechanism(s) (4, 6). We noticed that when CI27 was plated on LB agar for further protease-negative screening, there were small colony variants among large, parent-like colonies.

### Genome sequence comparison of CI27 WT and evolved protease-negative mutants

**Fig 2:**
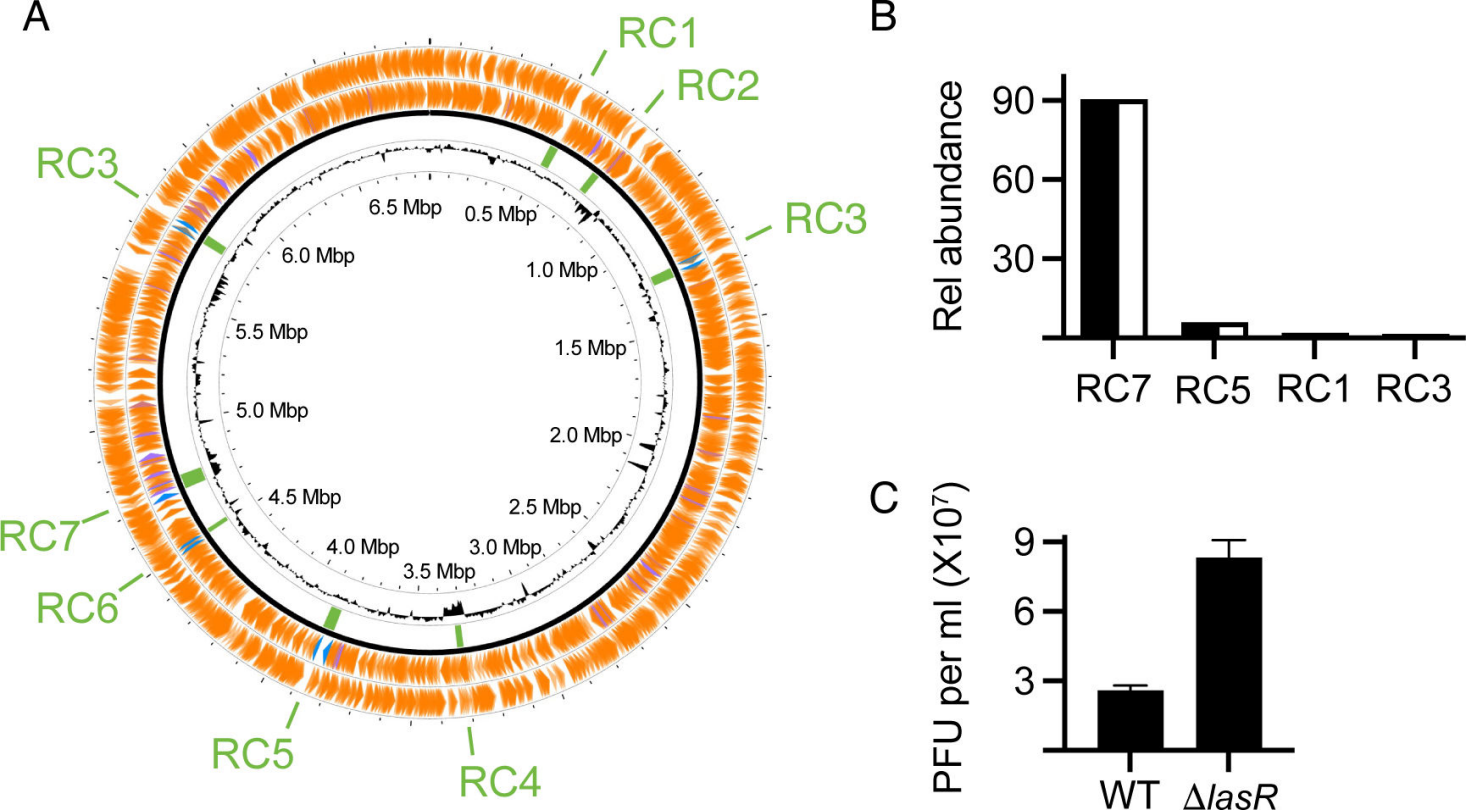
Genomic locations of CI27 prophages and phage particle levels in fluid from MMC-induced cultures. (**A**) Circular display of the CI27 genome, the locations of prophages 1–7 are shown in green. RC3 was found in two locations. In outer tracks, bacterial genes are in orange, and the blue and purple denote attachment sites and tRNAs, respectively. The inner track is GC content. (**B**) The relative abundance of phages in supernatant fluids of MMC-induced CI27 (black bars) and the CI27 *lasR* deletion mutant (white bars) as detected by RT-PCR. (**C**) Plaque-forming units in lysates of MMC-induced CI27 and the CI27 *lasR* deletion mutant. Plaquing was on the PAO1 RM^−^ strain. Note that the predominant virion detected by RT-PCR, RC7, does not form plaques on the indicator strain. Because RC7 constitutes about 90% of the virions, we estimate the phage titer to be close to 10^9^ per mL of lysate.

We re-sequenced and closed the CI27 genome. The deletion encompassing *hcnABC* is about 57 kb in length when compared to the PAO1 sequence and extends from PAO1 locus tag PA2164 to PA2221 (pseudomonas.com), where a prophage, we call RC4 (see below), is located in the CI27 genome (Fig. 2A).

To begin to understand how CI27 WT can restrain the emergence of LasR mutants in casein broth, we sequenced the genomes of three independent protease-negative isolates from a day-25 culture of an individual experiment. There was an identical *lasR* mutation in all three genomes. The mutant *lasR* allele encoded for a polypeptide with a V226I substitution. There was also a *psdR* mutation. The *psdR* gene encodes a repressor that, when mutated, allows more rapid growth on casein (8). Two of the three isolates also contained an SNP in *oprN*, which encodes the outer membrane porin of the MexEF-OprN efflux pump (9, 10). Mutations that inactivate the efflux pump lead to an enhanced RhlR-dependent activation of protease genes in PAO1 (9, 10). These CI27 LasR mutant genomes also contained SNPs in a gene annotated as *vgrG*, which encoded the Type VI secretion system Tip protein (11). In addition to the *psdR*, *oprN*, and *vgrG* mutations, we found a prophage, which we call RC5, present at a single location in the CI27 parent strain and at three additional locations in two of the three sequenced *lasR* mutant genomes. The RC5 genome indicates that it is an Mu-like transposable element. We note that, in addition to RC5, CI27 prophages RC1 and RC3 also have genes predicted to code for transposases (see below). We also found a small deletion in *pilC* in the third sequenced genome. The *pilC* gene codes for a protein required for assembly of type IV pili. The mutations in each isolate are shown in Table S1.

### Strain CI27 is a polylysogen with seven distinct phage genomes

We became interested in the census of prophages in the CI27 genome because the increased copy number of prophage RC5 in two of the mutant genomes suggested that transposition of the prophage might occur more frequently in LasR mutants than in the WT. Furthermore, there have been several recent publications on the relationship of *P. aeruginosa* QS to phage infection (12–14). In fact, we identified seven prophages (RC1–7) with one, RC3, found in two copies on the WT CI27 chromosome (Fig. 2A). The 36.7 kb RC1 is essentially identical to transposable *Pseudomonas* phage JBD69 (KU199708). RC3 (39.1 kb) is also a transposable prophage, and it is similar, but not identical, to RC1 and JBD25. A third putative transposable prophage is RC5 (38.7 kb). The closest relative of RC7 (57.4 kb) is *Pseudomonas* phage PP9W2, a member of the genus *Haihevirus*, but this prophage is sufficiently different to deserve classification into its own genus at the subfamily level with PP9W2. BLASTn against *P. aeruginosa* (taxid: 287) showed that prophages identical to RC5 and RC7 exist only in *P. aeruginosa* strain C127. Sequences identical to RC1 and RC3 occur in *P. aeruginosa* strains YTSY4 (CP054788) and PA942 (CP129200).

To determine which of the CI27 prophages produced phage particles, we induced phage production by mitomycin C (MMC) and analyzed the phage DNA in lysates by quantitative real-time polymerase chain reaction (qRT-PCR) with primers specific for each phage. We obtained qRT-PCR signals for RC1, RC3, RC5, and RC7, with RC7 being the most abundant of the group (Fig. 2B). We also constructed a *lasR* deletion mutant of CI27. Upon MMC induction of the CI27 LasR mutant, we detected the same phage in the same relative ratios as in lysates of the WT (Fig. 2B). We also used the lysates to enumerate plaque-forming units (PFUs) on PAO1 Δ*hsdRM*, a Type I restriction modification (RM^−^) mutant of PAO1 (15). As assessed by PCR, we detected plaques for RC1, RC3, and RC5 only. Presumably, PAO1 is restrictive for the most abundant phage induced by MMC, RC7. Interestingly, lysates of the CI27 LasR mutant contained approximately three times more PFUs than lysates of the WT (Fig. 2C).

**Fig 3:**
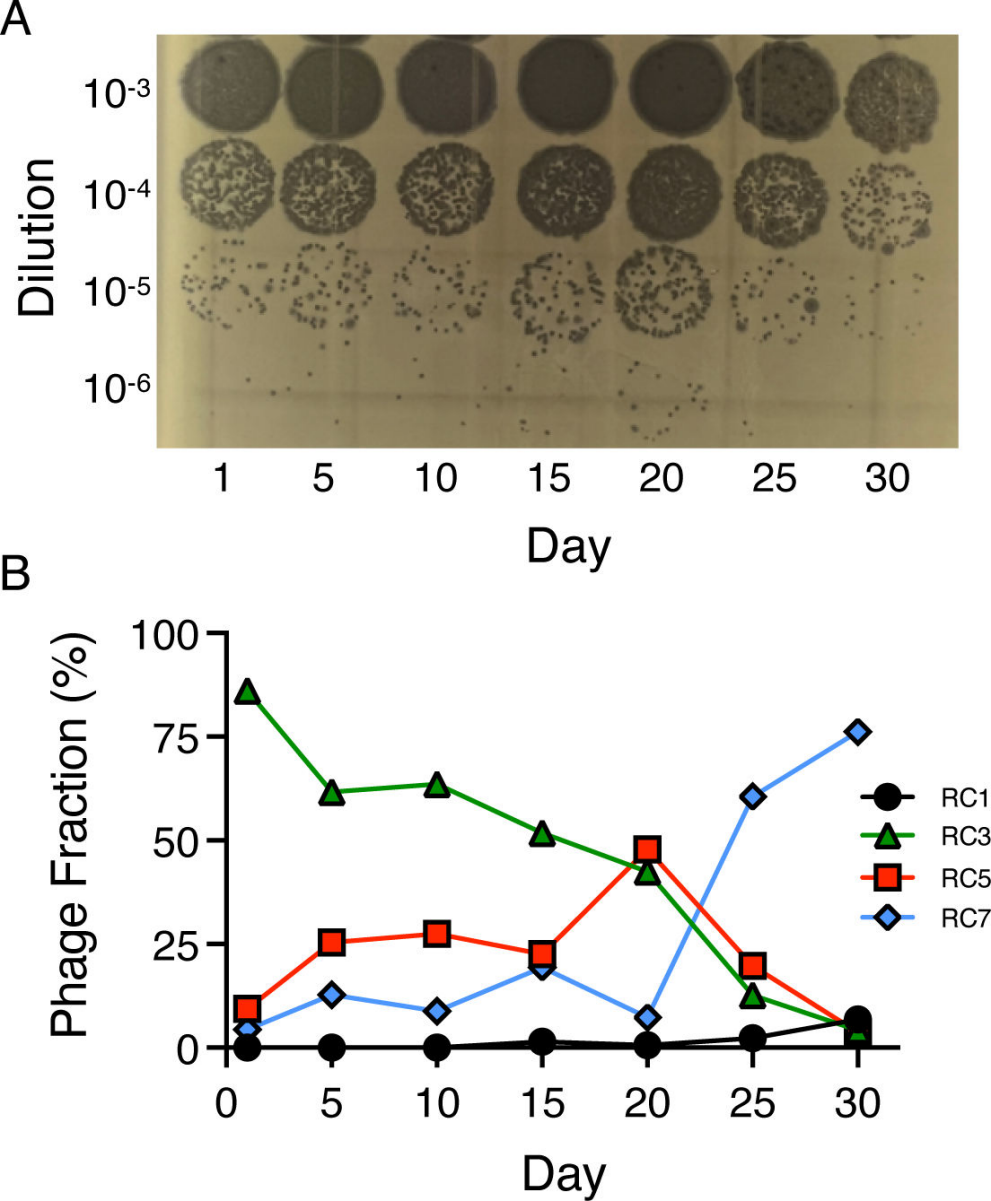
Spontaneous virion production by CI27 transferred daily in casein broth. (**A**) Plaque-forming units in CI27 in culture fluid at the end of growth on the day indicated. Ten microliters of tenfold diluted cell-free lysates were placed on the PAO1 RM^−^ mutant. (**B**) Relative abundance of virions in culture fluid as assessed by qRT-PCR.

If the phages harbored in the CI27 genome play any role in restricting the emergence of LasR mutants, we would expect to observe a high titer of virions in culture fluid in the evolution experiments. To test this idea, we examined phage abundance in CI27 casein broth supernatants at 5-day intervals with the PAO1 RM^−^ plaque assay (Fig. 3A). The abundance of phage as assessed by plaquing was relatively constant over the course of the experiment (about 10^8^–10^9^ per mL). RC7 was the most abundant phage in the MMC-induced lysates—but was it also present in the uninduced culture fluid? We tested the relative abundance of RC1, RC3, RC5, and RC7 in culture fluid throughout the evolution experiment using qRT-PCR. This experiment indicated that RC3 was the most abundant phage in the first half of passages. By day 25, RC7 had become the predominant phage (Fig. 3B).

### Phage lysis of the CI27 *lasR* deletion mutant

**Fig 4:**
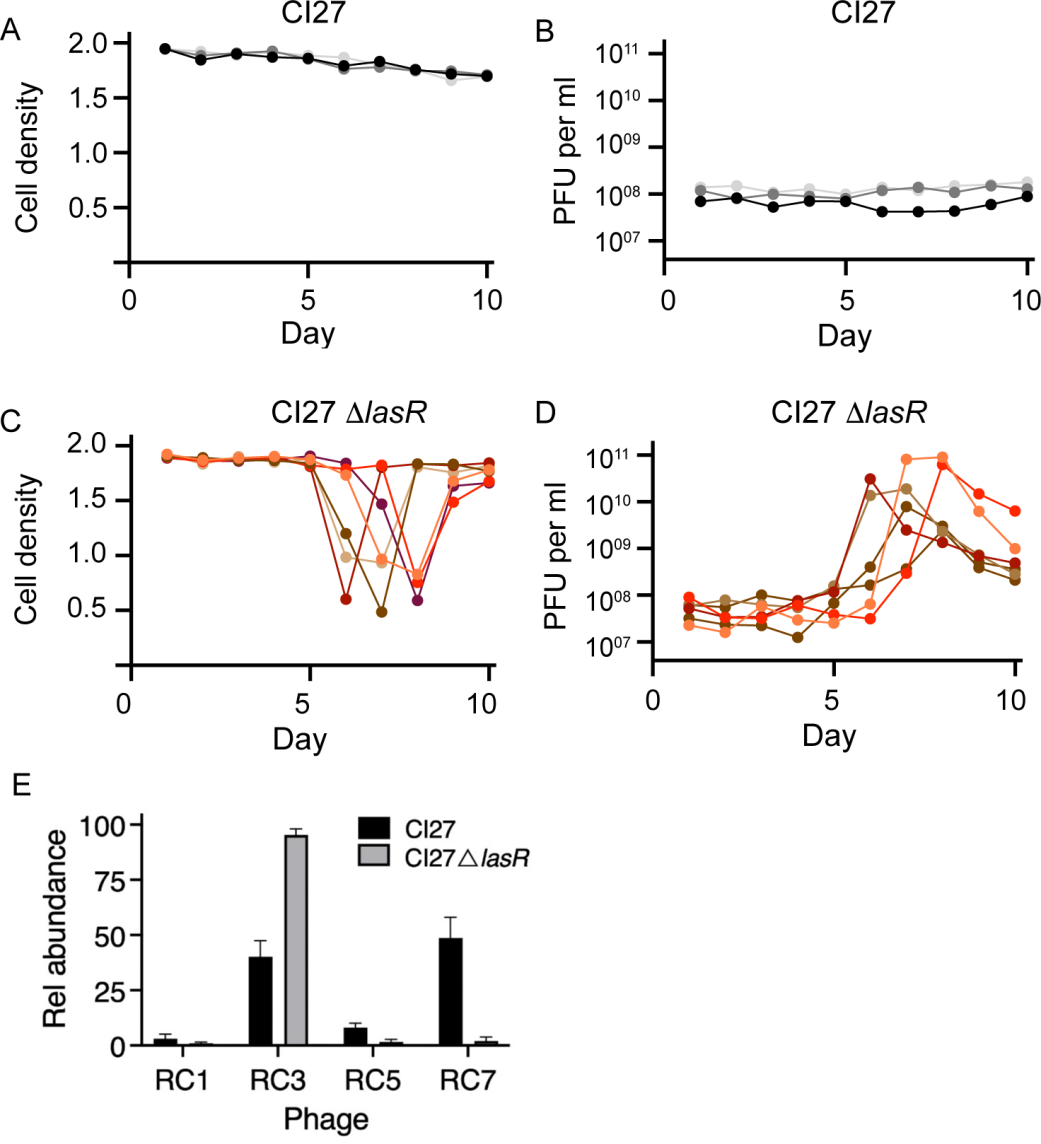
Prophage induction and cell lysis in the CI27 *lasR* deletion mutant. (**A**) Growth yield (OD_600_) of CI27 WT at the end of each growth cycle in casamino acids broth, and (**B**) the corresponding PFUs in the culture fluid. Panels **A and B** show three independent lineages. (**C**) Growth yield (OD_600_) of CI27 *lasR* mutant at the end of each cycle in casamino acids broth and (**D**) the corresponding PFUs in the culture fluid. Panels **C and D** show six independent lineages. Viable cell numbers of the lineages shown in panels A and C are in Fig. S4. (**E**) Relative abundance of virions as assessed by qRT-PCR of one of the LasR mutant lineages and for a WT lineage on the day the mutant lineage showed lysis.

Does LasR constrain prophage entry into a lytic cycle or play a role in phage immunity? To begin to address these questions, we used casamino acids broth to examine the growth of the CI27 *lasR* deletion mutant and CI27 WT (note: the *lasR* mutant requires co-culture with WT to grow in casein broth). Initially, the growth of the WT and mutant was similar. For the WT, growth and virion production remained relatively stable over the 10-day course of the experiment (Fig. 4A and B). However, the *lasR* deletion mutant cultures underwent a massive lysis around days 6 to 8, and the lysis event corresponded to a large increase in PFUs. Culture optical densities dropped roughly in half, and colony-forming units (CFU) were about 20% of the levels prior to the lysis event (Fig. 4C and D; Fig. S1). As assessed by qRT-PCR, spontaneously produced virions in WT cultures (after one day) were mainly RC7 and RC3, with lower levels of RC1 and RC5. In contrast, fluid from the lysed cultures of the LasR mutant contained almost exclusively RC3 (Fig. 4E). Apparently, RC3 replicates better in the LasR deletion mutant than in WT, and that viral loads in the culture fluid build to sufficient levels to overcome immunity in a large fraction of the population. These findings are consistent with the view that LasR is involved in constraining RC3 entry into a lytic state.

Investigations of strain PAO1 have shown that LasIR sits atop a hierarchical QS cascade. The LasIR system is required for activation of the RhlIR system and the non-AHL PQS QS system (16). To ask whether LasR was directly involved in constraining RC3, we asked whether the lysis event occurred in a RhlR mutant or a PqsR mutant (PqsR is required for Pqs signaling). We found that these CI27 QS mutants behaved similarly to the WT and did not undergo a lysis event (Fig. S2A). We also asked if a LasI signal synthase mutant was subject to the lysis event. If LasR interacts directly with a phage gene product to limit entry into the lytic state, then a LasI mutant might behave like the WT. Conversely, if LasR regulates expression of a factor involved in controlling or triggering RC3 entry into a lytic state, LasI should be required for the lysis event. Results with the CI27-*lasI* deletion mutant were nuanced: five of eight experiments with the LasI mutant showed a lysis event (Fig. S2B).

### Characterization of phage RC3 and related phages

**Fig 5:**
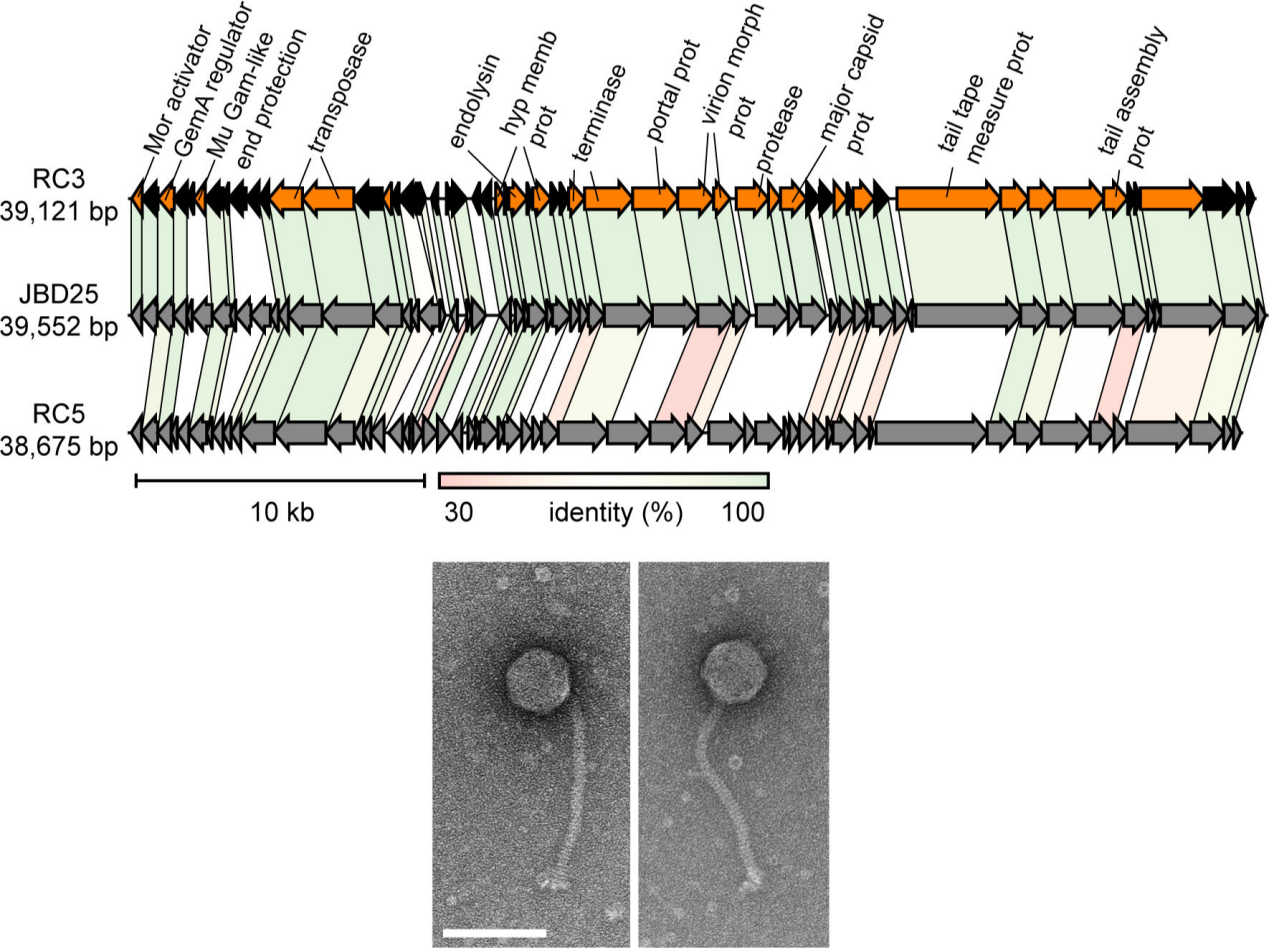
Genomes comparison of RC3, JBD25, and RC5 and electron micrographs of purified RC3. (Top) Alignments of the three phage genomes. The RC3 genes in color are those with predicted function, and genes for hypothetical proteins with unknown function are in black. Full annotation of the RC3 genes is shown in Table S2. The scale and percent identities among homologs in the genomes are below the RC5 genome. (Bottom) Electron micrographs of negatively stained RC3 virions. The marker bar is 100 nm.

We concentrated our efforts on RC3, and because RC5 is closely related to RC3, we included it in our genomic analysis. RC5 was also found in four copies in one of the evolved protease-negative bacterial genomes. These two prophages are closely related to JBD25, a Mu-like siphophage, which uses the *P. aeruginosa* Type four pilus (T4P) as a cell surface receptor (17). Alignments of RC3, RC5, and JBD25, including annotation of the RC3 genome, are shown in Fig. 5A and Table S2. There are only a few RC3 genes with no significant similarity to genes in the JBD25 genome. Electron microscopy showed that RC3 had a morphology like that of JBD25 with a long tail and an icosahedral head (Fig. 5B).

### LasR restricts lysis of other *P. aeruginosa* strains

**Fig 6:**
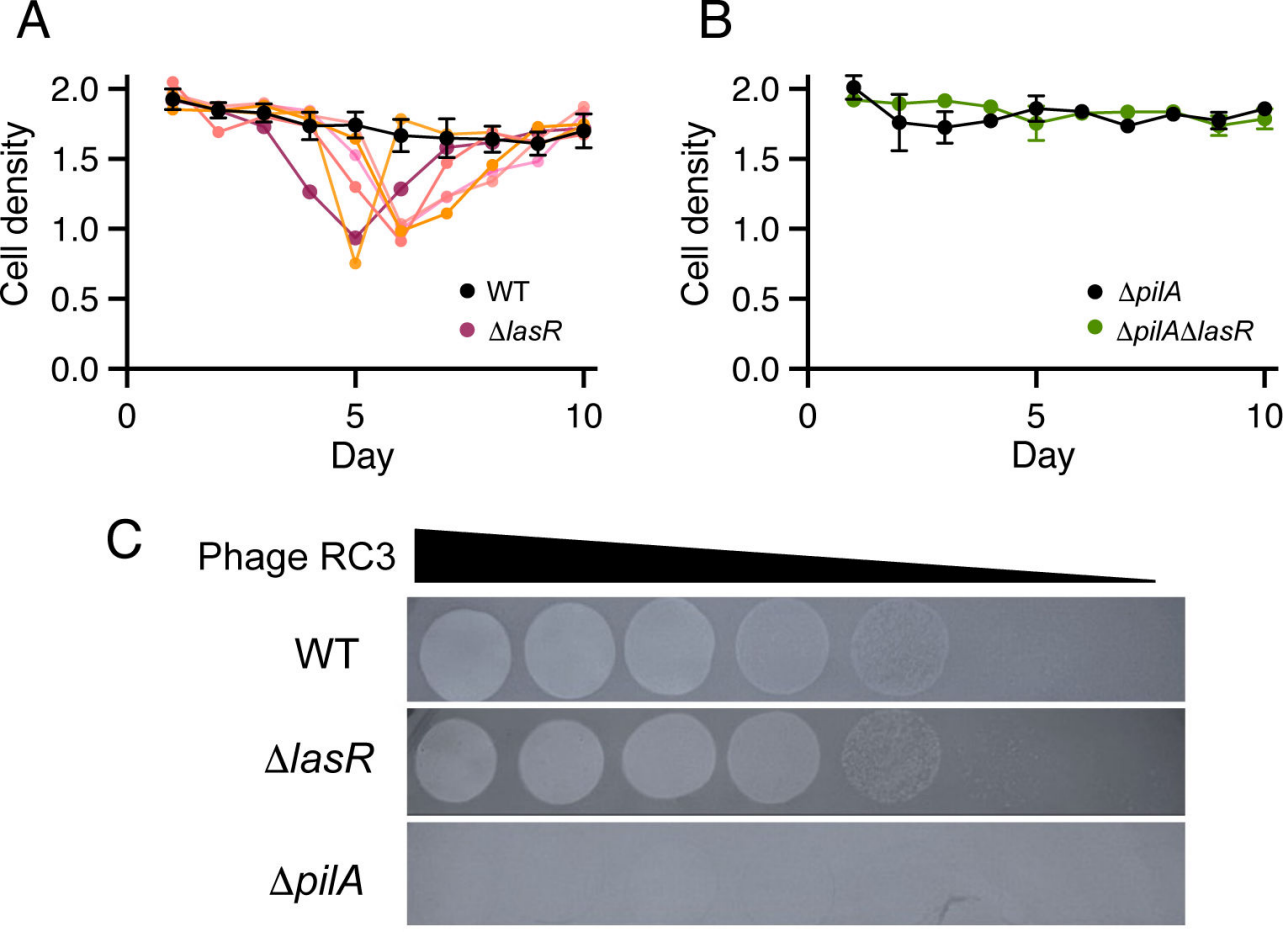
Subculturing in casamino acids broth and RC3 lytic infections. (**A and B**) RC3 lysogens were sub-cultured daily in casamino acids broth, and cell densities (OD_600_) at the end of each growth cycle are shown. (**A**) PAO1 RM^−^ and PAO1 RM^−^ Δ*lasR*. Results of six individual experimental lines are shown. For PAO1 RM^−^ (black), the means and standard errors of the means are shown. With PAO1 RM^−^ Δ*lasR*, the lysis events differed among the individual lines. Therefore, each is shown as a separated colored line. (**B**) PAO1 RM^−^ Δ*pilA* and a PAO1 RM^−^ Δ*pilA*Δ*lasR* double mutant. Results are means and standard errors of the means of six lineages. (**C**) RC3 lysis of PAO1 RM^−^ WT, the Δ*lasR* mutant, and the Δ*pilA* mutant. Tenfold dilutions of RC3 phage lysates (spotted left to right) on a lawn of PAO1 RM^−^ WT, the Δ*lasR* mutant, and the Δ*pilA* mutant as indicated. PAO1 RM^−^ WT and PAO1 RM^−^ Δ*lasR* appear susceptible to RC3 infection, and PAO1 RM^−^ Δ*pilA* appears resistant.

The lysis event observed with the CI27 LasR mutant correlated with a buildup of RC3 phage in the culture fluid. This suggests that LasR exerts some control over RC3 entry into a lytic state. To test this, we created RC3 lysogens of the PAO1 RM^−^ strain and another commonly studied strain, PA14, and generated *lasR* deletion mutants of these lysogens. The results were similar to those with CI27, the LasR mutants, but not the isolates with WT LasR showed the lysis event when transferred on casamino acids (Fig. 6; Fig. S3). Is this a specific characteristic of RC3 lysogens? To address this question, we lysogenized the RM^−^ strain with RC1, the closely related phage RC5 and JBD25, and constructed *lasR* deletion mutants of these lysogens. Neither of these showed a lysis event (Fig. S3). Apparently, LasR is required to limit the induction of RC3.

### An RC3 lysogen of the Δ*lasR* mutant shows impaired superinfection immunity

Phage RC3 did not plaque on the RM^−^ Δ*pilA* mutant (Fig. 6C), confirming T4P as the phage receptor. The RC3 titer displayed in the WT and *lasR* deletion mutants showed no difference, also suggesting that T4P is not regulated by LasR (Fig. 6C). We next tested whether RC3 superinfection is required for the lysis event by asking if the lysis occurred with a Δ*lasR*, Δ*pilA* double mutant of the RC3 lysogen transferred daily in casamino acids broth. This double mutant did not show the lysis event. This finding indicates that, regardless of a possible arms race between phage RC3 and the Δ*lasR* mutant, within a few passages, superinfection is needed for the lysis event. LasR facilitates population bacterial survival as RC3 titers increase (Fig. 6B). RC3 titers were determined daily by plaque assays. After the first three daily transfers, the phage titers in cultures of the Δ*lasR* RC3 lysogen were about 10–20% that of the WT lysogen (Fig. S4). Introduction of the Δ*pilA* mutation prevented superinfection and the lysis event and elevated RC3 titers during the initial three-day period (Fig. S4). Phage titers with the Δ*lasR* mutant increased dramatically over the next few days, except in the case of the Δ*pilA* mutant, indicating that superinfection is required for the increase in phage titers, and that the *lasR* mutation impaired the host’s ability to cope with the stress of RC3 phage superinfections. However, the mechanism of LasR-driven phage resistance remains unclear.

RT-PCR of the PAO1 RM^−^ RC3 lysogen genomic DNA showed a single copy of RC3, whereas there are two copies of RC3 in CI27. It seems possible that the extra copy of RC3 in the CI27 genome affords it an enhanced resistance to RC3 superinfection.

### An RC3 lysogen of a PAO1 RM^−^ cyanide mutant constrains infiltration by a LasR mutant

**Fig 7:**
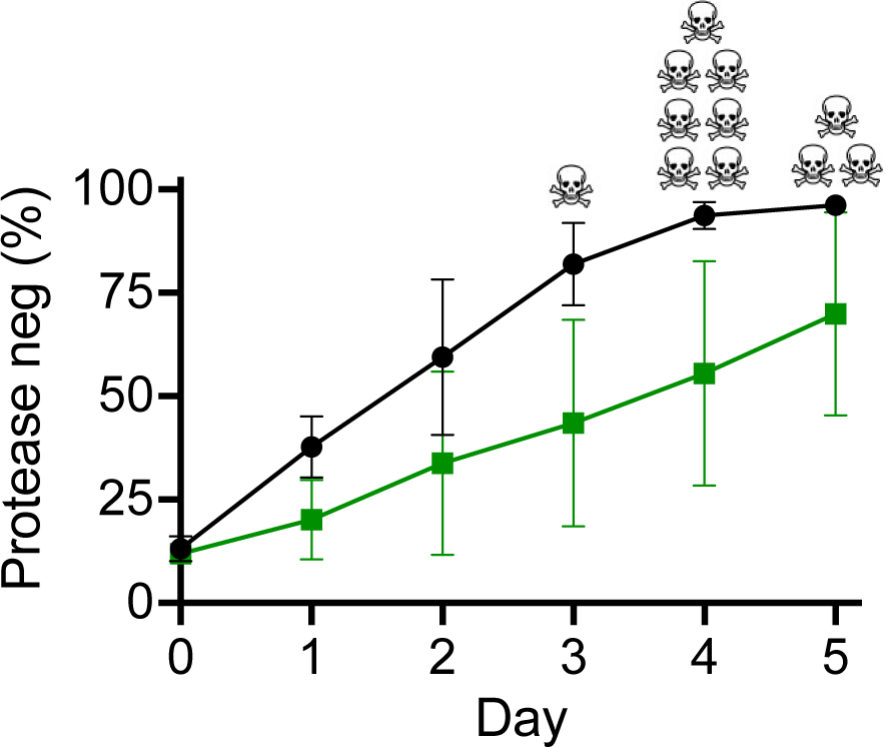
Competition between PAO1 RM^−^ WT Δ*hcnABC* (with or without RC3 prophage) and its *lasR* deletion mutant. The initial inocula consisted of the LasR WT at about 90% and LasR mutant at about 10%. Cells were transferred daily in casein broth. At the end of each day, the percent of *lasR* mutants (scored as protease-negative cells) was determined by plating clones on skim milk agar (see Materials and Methods). The green squares are the RC3 lysogen competitions, and the black circles are competitions with bacteria that were not lysogenized with RC3. Data points show means of 11 lineages, and the bars show standard errors of means. The black skull and crossbones indicate the day on which the co-cultures of the Δ*hcnABC* strains without RC3 did not grow on transfer. All of the competitions with the lysogens grew through the last transfer.

We investigated whether the ability of CI27 to constrain the emergence of LasR mutants was specific to CI27 or a more general feature of *P. aeruginosa* RC3 lysogens. To do this, we tested whether the PAO1 RM^−^ cyanide mutant lysogen can constrain LasR mutant siblings in casein broth co-culture. We started 11 cultures of the cyanide mutant RC3 lysogen with 90% LasR WT and 10% LasR mutant cells and 11 cultures that were not lysogenized (Fig. 7). Without RC3 lysogeny, the PAO1 RM^-^ LasR mutants invaded more rapidly than with RC3 lysogeny. Furthermore, without RC3 lysogeny, one culture ceased growth after the third transfer, seven ceased growth after the fourth transfer, and three ceased growth after the fifth transfer. None of the lysogen co-cultures ceased growing on transfer.

## DISCUSSION

Here, we describe a novel relationship between a prophage and its *P. aeruginosa* host. This relationship was revealed as we sought to understand how QS-cooperating populations constrain infiltration by QS mutants, which benefit from the cooperative activities of the group but do not pay the costs associated with cooperation. We discovered that a Mu-like transposable phage, we call RC3, can constrain competing LasR mutants of RC3 lysogens.

How the host regulates prophage entry into the lytic cycle, and how this benefits either the host or the prophage, is of interest. Recent reports have revealed intricate relationships between QS and temperate phages in *P. aeruginosa* (12–14) and in other bacterial species (18–20). Bacterial phage defense systems can be activated by QS, thereby protecting cells from phage. For *P. aeruginosa*, the interplay between QS and phage defense systems has been described. This interplay varies depending on the QS system and the phage encountered by the bacterium (21–25). Finally, some lysogenic phages have the ability to sense QS signals and thus use high host population density as a cue for entry into the lytic state (12). There are examples in which prophages inhibit *P. aeruginosa* QS by producing proteins that bind to and interfere with QS signal receptors (12, 26). Relevant to our study, others have found that *P. aeruginosa* lysogenized with two different ubiquitous phages can restrict infiltration by non-lysogenized QS mutants (14).

Growth of *P. aeruginosa* on casein requires QS activation of genes coding for extracellular proteases. These proteases are public goods, and QS mutants emerge as cheats during sequential transfer of cells in casein broth. The cheats cannot grow on their own in casein broth because they require the extracellular proteases produced by the WT (4). Previously, we reported that strain PAO1 constrains QS mutants and that this constraint involves QS activation of the *hcnABC* operon (6). We also showed previously that a different *P. aeruginosa* clinical isolate, CI27, has a chromosomal deletion encompassing *hcnABC* (1). Here, we show that this isolate can nevertheless constrain the emergence of QS mutants. Strain CI27 is a polylysogen with seven different prophage genomes. Upon MMC induction, CI27 LasR mutants produce about threefold more phage than WT. Apparently, QS suppresses the ability of the prophage to enter a lytic phase. We note that it has been demonstrated that two other temperate phages, D3112 and JBD30, replicate better in WT *P. aeruginosa* than they do in QS mutants (14). This is the opposite of the case with CI27.

We note that CI27 carries a relatively high number of prophage (seven distinct prophages, one of which is in two copies). Recent surveys indicate that most of the many sequenced *P. aeruginosa* genomes contain between one and six prophages (27, 28). Only one other isolate carries more than six prophages (29). We do not know if CI27 (and the other isolates) is particularly permissive for lysogeny, or if this might relate to the specific prophage carried or the order in which the infections occurred.

We found that after four or five daily transfers of a CI27 Δ*lasR* mutant in casamino acids broth, there is massive lysis, which corresponds to a burst in production of one of the lysogenic phages, RC3. The lysis event also occurs in RC3 lysogens of PAO1 RM^−^ Δ*lasR* mutants. Experiments with a PAO1 RC3 receptor mutant (Δ*pilA*) showed that superinfection was required for the lysis event. We do not favor the hypothesis that LasR is required for receptor gene activation. The RC3 receptor is the Type IV pilus, and Type IV pilus genes have not been reported to be activated by QS (21, 24, 25). As discussed above, there are reports of interplay between *P. aeruginosa* QS systems and anti-phage defense systems. We note that although CI27 has a battery of phage defense systems, PAO1 does not. PAO1 does have a restriction and modification system, which has been deleted in the mutant we used (PAO1 RM^−^). Because PAO1 RM^−^ can constrain LasR mutant siblings, it seems unlikely that LasR control of a phage defense system is involved in RC3*–P. aeruginosa* interactions.

It is reasonable to assume that RC3 can disadvantage LasR mutant cheats during growth on casein, because the mutants will be susceptible to phage reinfection, lysogen activation, or both. The susceptibility will be especially challenging for LasR mutants as they begin to emerge from WT populations during sequential transfer in casein broth, when relatively few mutant cells encounter many free phages.

In *P. aeruginosa*, the QS circuits are interrelated. The LasR mutation might affect phage via the RhlIR system or the PQS system (16). To address these possibilities, we constructed CI27 RhlR and PqsR mutants and found that they did not exhibit the massive lysis observed with the Δ*lasR* mutant. This indicates that LasR, or a factor regulated by LasR (other than PqsR or RhlR), interferes with the activation of RC3 and lysis. To attempt to discriminate between the possibility that LasR itself interacts with a phage polypeptide and the possibility that a LasR-regulated factor is involved in RC3 activation, we constructed a LasI QS signal generator mutant. Sometimes, but not always, this mutant exhibited the lysis event. From this result, we cannot determine whether LasR functions through the regulation of some factor, or whether cellular levels of LasR might be subtly influenced by the LasI mutation. There is precedence for the possibility that LasR itself might interact with an RC3 protein. The phage DMS3 protein Aqs1 binds to LasR and serves as a LasR anti-activator. Aqs1 also blocks superinfection by DMS3 (12). QS is active in CI27 (1), but it remains possible that there might be an RC3 LasR-binding protein. In this case, LasR would influence the activity of the phage protein rather than vice versa. Although we did not find an *aqs1* homolog in the RC3 genome, there may be another RC3 polypeptide or even an RNA, which binds to LasR.

As discussed above, the lysis event also occurs in RC3 lysogens of *P. aeruginosa* LasR mutants of strains other than CI27. *P. aeruginosa* lysogenized with RC1, RC5, or the RC3 relative JBD25 does not undergo the lysis event. There seems to be a specific interaction between LasR (or a LasR-regulated product) and RC3 that prevents the lysis event.

To test the idea that RC3 lysogeny can constrain the emergence of LasR mutants in the absence of cyanide policing, we performed competition experiments with cyanide mutants of strain PAO1 RM^−^ lysogenized with RC3, its *lasR* deletion mutant, and the mutant without RC3. In competitions without RC3, the LasR mutant outgrew the parent, and culture growth was not sustained by transfer five at the latest. In competitions between lysogenized WT and LasR mutants, growth was sustained through the last transfer. Our experiments support the conclusion that, at least in some conditions, LasR control of entry into the lytic cycle and stimulation of superinfection exclusion reduce the fitness of LasR mutants.

In terms of RC3 fitness (and the other phage in CI27), we imagine that QS-competent cells are better equipped to negotiate the stresses of high cell density and stationary phase than are QS mutants. In this situation, both host cells and their temperate phage might survive, whereas QS mutants may be less well equipped to survive stress, and entry into the lytic cycle prior to the demise of the host would provide benefit to the phage. We view the relationship as mutualistic. RC3 constrains the emergence of QS mutants, and QS provides a relatively stable environment for the RC3 lysogen.

## MATERIALS AND METHODS

### Bacterial strains and growth conditions

Bacteria used are described in Table S3. Unless otherwise specified, *P. aeruginosa* was grown in Luria-Bertani broth buffered with 50 mM 3-(N-morpholino) propane sulfonic acid, pH 6.8 (LB-MOPS broth). For evolution experiments, *P. aeruginosa* was grown in PM broth with 1% of casamino acids or sodium caseinate as the sole source of carbon and energy (30, 31). *Escherichia coli* was grown in LB broth. For colony growth, we used LB agar (1.5% agar), milk agar plates (4), or caseinate agar plates (31), as indicated. Broth cultures were grown at 37°C with shaking at 220 rpm. Agar plates were incubated at 37°C as described previously (31). Plate counting on LB agar was used to determine bacterial CFUs, and phage PFUs were determined by using the agar overlay method described below.

### Construction of *P. aeruginosa* mutants

Mutants with deletions of *lasR*, *lasI*, *rhlR*, *pqsR*, and *hcnABC* were derived from CI27 or from the PAO1 RM^−^ strain by a homologous recombination-based two-step allelic exchange approach (32). Briefly, about 500 bp of DNA flanking the gene of interest were PCR-amplified and cloned in pEXG2 by using *E. coli*-mediated DNA assembly (33) or Gibson assembly (New England Biolabs). The primers used to generate the pEXG2 knockout plasmids are listed in Table S4. *E. coli* S17-1 or *E. coli* DH5α with helper PRK2013 were used to deliver knock-out plasmids to *P. aeruginosa* via conjugation. Merodiploids were selected by plating on Vogel-Bonner Minimal Medium agar (34) with 100 µg/mL gentamicin, and deletion mutants were then selected on LB (without added salt) agar containing 10–15% sucrose. All deletion mutations were confirmed by PCR of genomic DNA.

### Evolution and competition experiments

To initiate evolution experiments, we used 50 µL of an overnight LB-MOPS culture to inoculate 3 mL casamino acid broth (in 16 mm tubes) or casein broth (in 18 mm tubes). Cells were grown in the indicated broth for 24 h, and then, the cultures were used to inoculate fresh broth (inoculum size, 50 µL). For experiments in casamino acid broth, we measured cell density (OD_600_), CFUs, and PFUs, as indicated. For experiments in casein broth, we determined abundance of protease-negative cells by plating 100 isolated colonies on milk agar as described elsewhere (4). Competition experiments were performed in a similar fashion, except that the starting inocula were from overnight cultures to give a ratio of 90% LasR-positive to 10% LasR-negative cells.

### Genome analyses

Genome sequencing and annotation were performed commercially by Novogene. We used PHASTEST (https://phastest.ca/) for phage lysogen prediction and both PHASTEST and HHpred (https://toolkit.tuebingen.mpg.de/tools/hhpred) for annotation.

### Prophage induction, plaque assays, and measurements of relative abundance of phage particles

For MMC induction, we used an overnight culture of *P. aeruginosa* CI27 as inoculum (1%) at an OD_600_ of 1. We added MMC to a final concentration of 1 µg/mL. The MMC-treated cultures were incubated an additional 5 h, at which time lysis was evident. Lysates were sterilized using 0.22 µm pore size polyether sulfone syringe filters, and the filtrates were stored at 4°C. For spontaneous lysis, cultures were centrifuged, and the supernatant fluid was filter-sterilized for further use.

Double-layer plates were used for general plaque assays. Briefly, 40 µL of a fresh recipient bacterial culture (OD_600_ about 0.8) was added to 4 mL soft agar (LB with 0.6% [wt/vol] agar) at 45°C, and the mixture was spread on a base plate (LB with 1.5% agar). For phage purification, single plaques grown from lysates were picked and plaque-purified three times before verifying the phage by PCR with indicator primers. Purified phage suspensions were diluted to an appropriate titer and applied to a double-layer plate for plaquing. We used SM buffer (1 mM MgSO_4_, 4 mM CaCl_2_, 50 mM Tris-Cl, 100 mM NaCl, pH 8) (13) for dilutions and phage suspensions.

DNA in cell-free lysates or supernatants was extracted with a Puregene DNA Extraction Kit (Qiagen). The concentration of DNA was normalized to 2 µg per μL for qRT-PCR experiments. Phage-specific primers are listed in Table S4. The signal of phage-specific primers indicated the phage and prophage genome amounts, and *P. aeruginosa* gene primers for *rhlR*, *pqsR*, and *phzE* were used to measure residual gDNA in the lysate. In this way, we were able to sum up the phage totals and assess the relative abundance of different phage particles. The qRT-PCR reactions (20 µL) contained 20 ng total DNA, 500 nM of each primer pair, and 1× Universal SYBR Green Supermix (Bio-Rad). All primer sets were used with the same cycling conditions: 95°C for 10 min, followed by 40 cycles of 95°C for 10 s, 60°C for 15 s, and 72°C for 20 s. Phage DNA copy numbers were quantified from DNA samples in triplicate by using a Bio-Rad CFX96 Real-Time System.

### Creation of phage lysogens

To create phage lysogens of the PAO1 RM^−^ strain and strain PA14, we mixed plaque-purified phage and bacteria in soft agar and spread the mixtures on base agar plates. After incubation for 36–48 h, plaques were apparent, and bacterial cells within a plaque were picked and re-streaked three times. Lysogeny was confirmed by PCR with bacterial genomic DNA as template and phage genome primer pairs.

### Transmission electron microscopy

Transmission electron microscopic imaging was performed at Fred Hutchinson Cancer Research Center Electron Microscopy Shared Resource Facility (Seattle, WA). Purified phage suspensions (1 × 10^8^ to 1 × 10^9^ PFU per mL) were concentrated using Millipore Ultra Centrifugal Filter (100 kDa MWCO) centrifugation at 2,000 × *g*, and phages were resuspended in 2% uranyl acetate. The negatively stained phage particles were imaged on a TFS L120C Talos microscope. The microscope was operated at 120 kV with a magnification range of 10,000–60,000 to assess particle size and distribution. All images were recorded on a Thermo Fisher Scientific Ceta CMOS high-resolution 16M camera.

### Hydrogen cyanide assays

Cyanide was detected by using cyanogenic paper as described elsewhere (35). Cells were grown overnight in LB-MOPS and used to spot inoculate peptone agar plates. After 12 h at 37°C, the plates were overlayed with the cyanogenic paper, which turned blue when exposed to cyanide.

## Data Availability

The *P. aeruginosa* CI27 genome sequence data have been deposited at NCBI (accession number PRJNA600039, GCF_022810825.1).

## SUPPLEMENTAL MATERIAL

Supplemental Figures and Tables mbio.02360-25-s0001.pdf Fig. S1-S4 and Tables S1-S4.

ASM does not own the copyrights to Supplemental Material that may be linked to, or accessed through, an article. The authors have granted ASM a non-exclusive, world-wide license to publish the Supplemental Material files. Please contact the corresponding author directly for reuse.
