# Supplementary material for: Collaboration between a temperate phage and Pseudomonas aeruginosa quorum sensing constrains social cheats
Source: mBio. 2025 Sep 22;16(11):e02360-25. doi: 10.1128/mbio.02360-25 (PMC12607904; doi:10.1128/mbio.02360-25)
Supplement: Supplemental Figures and Tables — Fig. S1-S4 and Tables S1-S4. [file mbio.02360-25-s0001.pdf]

Collaboration between a temperate phage and *Pseudomonas aeruginosa* quorum sensing constrains social cheats

Ruiyi Chen[1], Beth Traxler[1], Andrew M. Kropinski[2] and E. Peter Greenberg[1]
[1]Department of Microbiology, University of Washington School of Medicine, Seattle, WA 98195
[2]Department of Pathobiology, Ontario Veterinary College, University of Guelph, Guelph, Canada

SUPPLEMENTAL FIGURES and TABLES (4 Figures and 4 Tables)

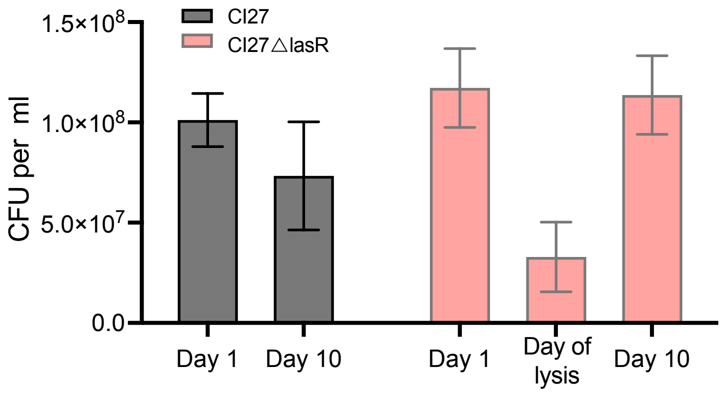

Figure S1. Cell numbers (CFU) of CI27 Δ*lasR* and WT transferred daily in casamino acids broth. The Day-1, Day-10, and the day of lysis events (Day-lyse) in lines of Δ*lasR* mutant are shown. For CFU determinations cultures were sampled just prior to transfer to fresh broth. The data are means of 3 WT lineages and 6 Δ*lasR* lineages. Bars show standard errors of means. The WT did not exhibit a lysis event.

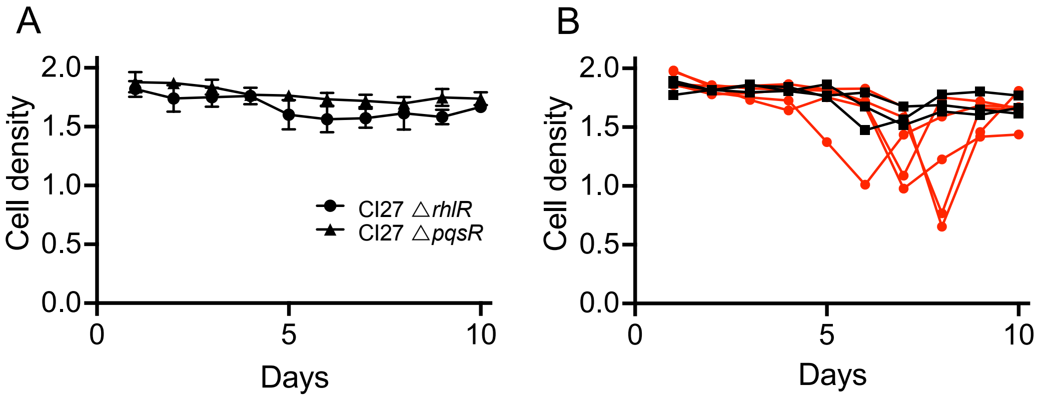

Figure S2. Cell yields (OD_{600}) at the end of each daily transfer of CI27. (A) *lasI* deletion mutant. (B) *rhlR* or *pqsR* deletion mutants. The arrows in panel A mark the days when cell lysis occurred in 5 of 8 lineages (the red lines). A lysis event was not observed in the other three lineages (black lines). In B, the results are the means of 6 lineages of the *rhlR* deletion mutant and 5 lineages of the CI27 *pqsR* deletion mutant. The bars indicate standard errors of the means.

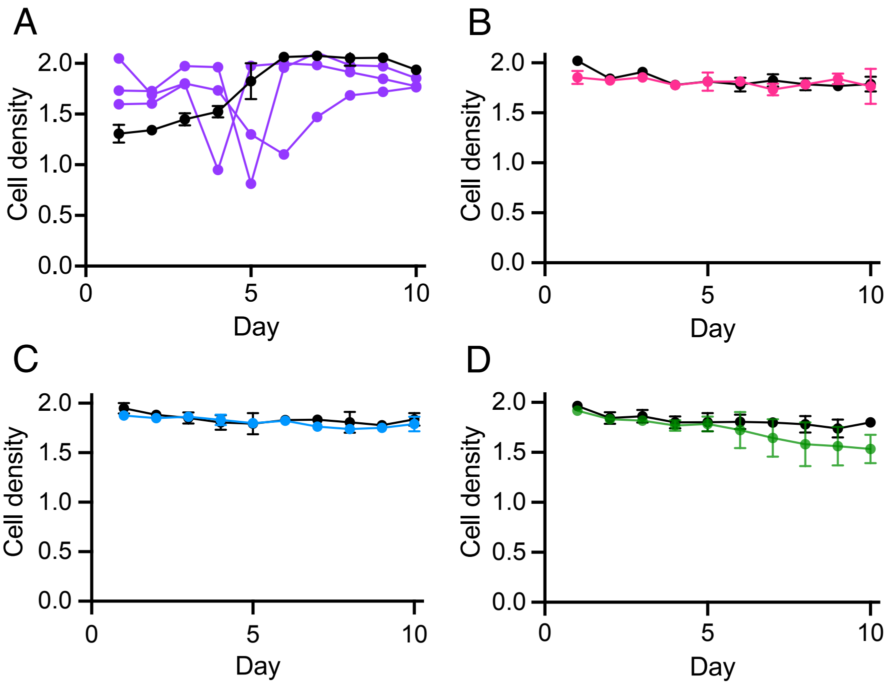

Figure S3. A *lasR* deletion mutant of a *P. aeruginosa* PA14 RC3 lysogen shows the lysis event and RC1, RC5 and JBD25 lysogens of a *P. aeruginosa* PAO1 RM$^-$ *lasR* deletion mutant do not exhibit the spontaneous lysis event. (A) Daily transfer of PA14 RC3 (black line) and PA14 RC3 Δ*lasR* (colored lines). (B) Δ*lasR* mutants of PAO1 RM$^-$ RC1 lysogen, (C) RC5 lysogen and (D) JBD25 lysogen. Cell density was determined as CFUs per ml at the end of each daily cycle. Results are means of six experimental lines with the exception of WT PA14 RC lysogen (three independent lines). In cases where there was not a lysis event results are shown as means and the bars show the standard errors of the means.

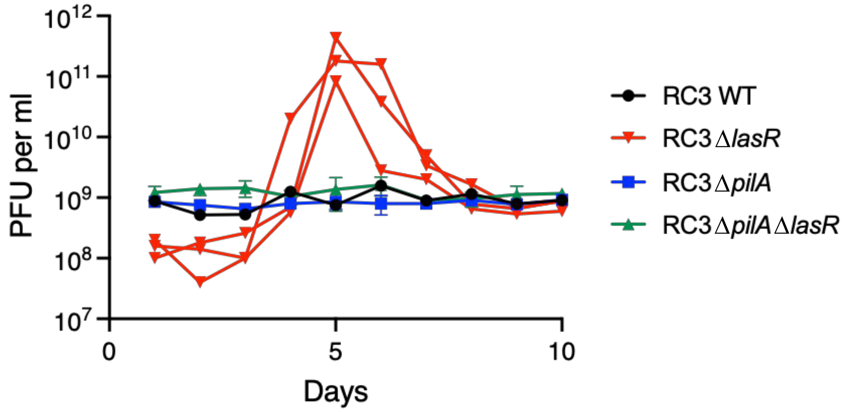

Figure S4. The PFUs in culture fluid of RM⁻ RC3 lysogens transferred daily in casamino acids broth. The results of RM⁻ RC3, PAO1 RC3 *pilA,* and PAO1 RC3 *pilA lasR* were the means of 3 lineages, and the bars show the standard error of the mean. Curves of RM⁻ RC3 Δ*lasR* replicas were shown independently.

Table S1. Mutations in evolved protease-negative CI27 isolates

Isolate	Mutation Type	PA gene_ID	Annotation
1	Deletion_3 bp	PA4527_pilC	Type 4 fimbrial assembly protein, PilC
	Nonsynonymous_SNP	PA1430_lasR	Quorum sensing transcription regulator, LasR
	Nonsynonymous_SNP	PA4499_psdR	*Pseudomonas* dipeptide regulator, PdsR
2	Deletion_30 bp	PA2304_ambC	Putative regulatory protein; TauD/TfdA family dioxygenase
	Nonsynonymous_SNP	PA1430_lasR	Quorum sensing transcription regulator LasR
	Nonsynonymous_SNP	PA4499_psdR	*Pseudomonas* dipeptide regulator, PdsR
	Nonsynonymous_SNP	PA1511_VgrG2a	Type VI secretion system tip protein VgrG
	Nonsynonymous_SNP	PA4501_opdP	Outer membrane porin, OprD family
3	Nonsynonymous_SNP	PA1430_lasR	Quorum sensing transcription regulator, LasR
	Nonsynonymous_SNP	PA4499_psdR	*Pseudomonas* dipeptide regulator, PdsR
	Nonsynonymous_SNP	PA1511_vgrG2a	Type VI secretion system tip protein VgrG
	Nonsynonymous_SNP	PA4501_opdP	Outer membrane porin, OprD family

Table S2. RC3 genome annotation and similarity to JBD25 and RC5

RC3		JBD25			RC5		
Protein ID	Annotation[a]	Protein ID	Identity	Similarity	Protein ID	Identity	Similarity
WP_003127781.1	**Mor transcription activator**	YP_009168726.1	1	1	WP_003094179.1	0.18	0.24
WP_003117363.1	hypothetical protein	YP_009168727.1	1	1			
WP_003117362.1	**regulatory protein GemA**	YP_009168728.1	0.99	1	WP_023657063.1	0.8	0.86
WP_003117361.1	hypothetical protein	YP_009168729.1	0.97	0.99	WP_003129239.1	0.99	1
WP_003117360.1	hypothetical protein				WP_003117360.1	1	1
WP_003142313.1	**Mu Gam-like end protection**				WP_003142313.1	1	1
WP_003094190.1	hypothetical protein	YP_009168732.1	0.95	0.95	WP_031638066.1	0.99	1
WP_010791825.1	hypothetical protein	YP_009168733.1	0.97	0.97	WP_033990702.1	0.94	0.95
WP_010791824.1	hypothetical protein				WP_003094193.1	0.54	0.56
WP_010791823.1	hypothetical protein						
WP_003142274.1	hypothetical protein	YP_009168737.1	0.97	0.98	WP_003094197.1	0.96	0.97
WP_003142272.1	**transposase B**	YP_009168738.1	0.99	0.99	WP_003094200.1	0.98	0.99
WP_128688208.1	**transposase A**	YP_009168739.1	0.98	0.99	WP_033996663.1	0.97	0.98
WP_003094208.1	hypothetical protein	YP_009168740.1	1	1	WP_023101705.1	0.87	0.92
WP_003094211.1	**IclR family transcriptional regulator**	YP_009168741.1	1	1	WP_003142307.1	0.94	0.97
WP_003094213.1	hypothetical protein	YP_009168742.1	1	1	WP_003142306.1	0.95	0.97
WP_023127890.1	hypothetical protein	YP_009168743.1	0.63	0.63	WP_010791820.1	0.92	0.93
WWQ72262.1	hypothetical protein						
MBF3267794.1	hypothetical protein	YP_009168744.1	0.87	0.87			
WP_003094216.1	**DNA-binding protein**	YP_009168745.1	1	1	WP_003142304.1	0.93	0.95
WP_023127888.1	hypothetical protein	YP_009168746.1	0.98	0.98	WP_025921031.1	0.39	0.42
WP_031299624.1	**putative lipoprotein**	YP_009168747.1	0.96	0.96	WP_124084312.1	0.95	0.95
WP_124084312.1	hypothetical protein						
WP_029981997.1	hypothetical protein	YP_009168748.1	1	1	WP_029981997.1	1	1
WP_023465062.1	**hypothetical membrane protein**	YP_009168749.1	1	1	WP_033868850.1	0.98	0.98
WP_003094227.1	hypothetical protein	YP_009168750.1	1	1	WP_162832300.1	0.96	0.98
WP_003138153.1	**endolysin**	YP_009168751.1	1	1	WP_034011213.1	0.99	0.99
YP_009168752.1	hypothetical protein	YP_009168752.1	1	1	YP_010773912.1	0.93	0.97

WP_003138155.1	**hypothetical membrane protein**	YP_009168753.1	1	1	WP_033868849.1	0.64	0.72
WP_003117315.1	hypothetical protein	YP_009168754.1	1	1			
WP_003121465.1	hypothetical protein	YP_009168755.1	0.99	0.99			
WP_003121466.1	**terminase, small subunit**	YP_009168756.1	0.99	0.99	WP_003139991.1	0.44	0.58
WP_003138156.1	**terminase, large subunit**	YP_009168757.1	0.99	1	WP_003139988.1	0.81	0.86
WP_023093201.1	**portal protein**	YP_009168758.1	0.95	0.96			
WP_003138159.1	**virion morphogenesis protein**	YP_009168759.1	1	1	WP_003139984.1	0.36	0.47
WP_023127646.1	**virion morphogenesis protein**	YP_009168760.1	0.99	0.99	WP_003139982.1	0.49	0.61
WP_003138161.1	**protease**	YP_009168761.1	0.99	0.99			
WP_003121593.1	**virion structural protein**	YP_009168762.1	1	1			
WP_003127513.1	**major capsid protein**	YP_009168763.1	1	1			
WP_015649419.1	hypothetical protein	YP_009168764.1	0.71	0.71			
WP_003121492.1	hypothetical protein	YP_009168765.1	1	1	WP_003094261.1	0.47	0.67
WP_003138162.1	**virion structural protein**	YP_009168766.1	0.99	0.99	WP_003094263.1	0.52	0.7
WP_003127509.1	hypothetical protein	YP_009168767.1	1	1	WP_003139972.1	0.42	0.59
WP_016852449.1	**virion structural protein**	YP_009168768.1	0.99	0.99	WP_003094267.1	0.55	0.73
WP_016852450.1	hypothetical protein	YP_009168769.1	0.99	0.99	WP_003139971.1	0.43	0.57
WP_033868716.1	**tail tape measure protein**	YP_009168771.1	0.93	0.95			
WP_003119570.1	**virion structural protein**	YP_009168772.1	0.99	0.99	WP_218821102.1	0.96	0.98
WP_003142236.1	**virion structural protein**	YP_009168773.1	0.94	0.98	WP_003139965.1	0.91	0.94
WP_243597502.1	**virion structural protein**	YP_009168774.1	0.97	0.98	WP_003139962.1	0.64	0.75
WP_049885375.1	**tail assembly protein**	YP_009168775.1	0.99	1	WP_003139961.1	0.39	0.55
WP_003094581.1	hypothetical protein	YP_009168776.1	0.97	0.99			
WP_023102217.1	hypothetical protein	YP_009168777.1	0.96	0.99			
WP_003138165.1	**virion structural protein**	YP_009168778.1	0.96	0.97	WP_003139959.1	0.51	0.68
WP_003121471.1	hypothetical protein	YP_009168779.1	0.96	0.97	WP_003139957.1	0.83	0.9
WP_003121472.1	hypothetical protein	YP_009168780.1	0.97	0.98	WP_003139956.1	0.91	0.95
WP_003121473.1	hypothetical protein				WP_015649550.1	0.76	0.81

[a]Gene annotation for RC3 was added by the NCBI Prokaryotic Genome Annotation Pipeline (PGAP), fine-tuned by PHASTEST, HHpere and AlphaFold.

Table S3. Strains used

Strain	Relevant characteristic	Reference or source
E. coli		
DH5α	*fhuA2Δ(argF-lacZ)U169 phoA glnV44 Φ80Δ(lacZ)M15 gyrA96 recA1 relA1 endA1 thi-1 hsdR17*	NEB Catlog#C2987H
PRK2013	*ori* colE1, RK2 derivative, Kanr, *mob*+, *tra*+	Figurski and Helinski, 1979
P. aeruginosa		
PAO1	PAO1 wild-type	Wang *et al.*, 2015
PAO1 Δ*hcnC*	PAO1 *hcnC* deletion mutant	Wang *et al.*, 2015
CI27	Wild-type strain	Chugani *et al.*, 2012
CI27 Δ*lasR*	CI27 *lasR* deletion mutant	This study
CI27 Δ*lasI*	CI27 *lasI* deletion mutant	This study
CI27 Δ*rhlR*	CI27 *rhlR* deletion mutant	This study
CI27 Δ*pqsR*	CI27 *pqsR* deletion mutant	This study
PAO1 RM$^-$	PAO1 *hdsM* deletion mutant, restriction-modification deficient strain	Mendoza *et al.*, 2020
PAO1 RM$^-$ Δ*lasR*	PAO1 RM$^-$ *lasR* deletion mutant	This study
PAO1 RM$^-$ Δ*pilA*	AO1 RM$^-$ *pilA* deletion mutant	This study
PAO1 RM$^-$ RC3	PAO1 RM$^-$ RC3 lysogen	This study
PAO1 RM$^-$ RC3 Δ*lasR*	PAO1 RM$^-$ RC3 lysogen *lasR* deletion mutant	This study
PAO1 RM$^-$ RC1	PAO1 RM$^-$ RC1 lysogen	This study
PAO1 RM$^-$ RC1 Δ*lasR*	PAO1 RM$^-$ RC1 lysogen *lasR* deletion mutant	This study
PAO1 RM$^-$ RC5	PAO1 RM$^-$ RC5 lysogen	This study
PAO1 RM$^-$ RC5 Δ*lasR*	PAO1 RM$^-$ RC5 lysogen *lasR* deletion mutant	This study
PAO1 RM$^-$ JBD25	PAO1 RM$^-$ JBD25 lysogen	This study
PAO1 RM$^-$ JBD25 Δ*lasR*	PAO1 RM$^-$ JBD25 lysogen *lasR* deletion mutant	This study
PAO1 RM$^-$ RC3 Δ*pilA*	PAO1 RM$^-$ RC3 lysogen *pilA* deletion mutant	This study
PAO1 RM$^-$ RC3 Δ*pilA*Δ*lasR*	PAO1 RM$^-$ RC3 lysogen *pilA* and *lasR* deletion mutant	This study
PAO1 RM$^-$ RC3 Δ*hcnABC*	PAO1 RM$^-$ RC3 lysogen *hcnABC* deletion mutant	This study
PAO1 RM$^-$ RC3 Δ*hcnABC*Δ*lasR*	PAO1 RM$^-$ RC3 lysogen *hcnABC* and *lasR* deletion mutant	This study

Table S4. Primers used

Primer	Sequence (5' → 3')	Note
For qRT-PCR		
RC1-markA-F	GGGGAAATAGAGGTAGCCGC	Phage/prophage RC1 qPCR markers
RC1-markA-R	TACTGGTGGTGCATTACGCC	
RC1-markB-F	TCGTGCCGAACCTCGTTATG	
RC1-markB-R	CCATATCCAAGGCGGTCCTC	
RC2-mark-F	CGTCTCACCAGGGTTATCGG	Phage/prophage RC2 qPCR marker
RC2-mark-R	ATTCTCACCCGTGGATACCG	
RC3-markA-F	ACTTCTTCGACACCGACCAC	Phage/prophage RC3 qPCR markers
RC3-markA-R	CGTTTTCGTCTTTCTCGGCG	
RC3-markB-F	AGCAGTACATCACCGATGGC	
RC3-markB-R	GTCAGTTGGAAAGTGGCTGC	
RC4-mark-F	ACGATTCGGGAAGGCTTTGG	Phage/prophage RC4 qPCR marker
RC4-mark-R	GGGCTTACGGAGAAGCAACT	
RC5-markA-F	GACGAACTACAACCCCCTGG	Phage/prophage RC5 qPCR markers
RC5-markA-R	CAGTGTCCTTTTGTTGCCCG	
RC5-markB-F	TGCTCTTCGGCATCGCTATT	
RC5-markB-R	CGAGAAGGACAAGTTCGGCT	
RC6-mark-F	TTGATTGGCCGTAGCAACCT	Phage/prophage RC6 qPCR marker
RC6-mark-R	TTTCAATCTCGTCCGCTCCC	
RC7-markA-F	CCTCTCTGAAGGGCGATGTT	Phage/prophage RC7 qPCR markers
RC7-markA-R	CCTTTACGCTTGCATACGCC	
RC7-markB-F	GTCCTTCAGGGTGTTGAGGG	
RC7-markB-R	ACCTGTTGAAGGACTCTGCG	
rhlR-mark-F	ATTTGCTCAGCGTGCTTTCC	Single copy chromosome gene markers
rhlR-mark-R	TCAGCTTCTGGGTCAGCAAC	
pqsR-mark-F	GCTTCGCCTGATCCCTTACA	
pqsR-mark-R	CTCACCGTATCGCAGAACGA	
phzE-mark-F	CGTCGGCTTCTACAACACCT	Double copies chromosome gene marker
phzE-mark-R	GAATTGCATGGAGGCGAAGC	
For plasmid construction/ gene knock-out		
lasR-up-F	CCTTGAACACTTGAGCACGC	For constructing the pEXG2-*lasR* KO plasmid and Δ*lasR*$_{69\text{-}651\ bp}$
lasR-up-F	AAGTTCGGTGTGACCTCCCG	
lasR-down-F	TGCAGGATGGCGCTCCACTC	
lasR-down-R	AAAGTGGCTATGTCGCCGGG	
lasI-up-F	ATGGCCGTTAATTTGGGTCT	For constructing the pEXG2-*lasI* KO plasmid and Δ*lasI*$_{77\text{-}540bp}$
lasI-up-F	TGCGCTCCTTGAACACTTGA	
lasI-down-F	AATGCCAAGACCCAGATCGC	
lasI-down-R	CCTGATCGGCAACCTTACCC	

pqsR-up-F	ACCTCCAAAACGACGACTCC	For constructing the pEXG2-*pqsR* KO plasmid and △*pqsR*$_{182\text{-}983bp}$
pqsR-up-F	GCATGTAAGGGATCAGGCGA	
pqsR-down-F	CGCCGCACCAGAGTAGAG	
pqsR-down-R	ATACGCCGTTGCCGACGAT	
rhlR-up-F	GTCCATCCGGGCGGTATC	For constructing the pEXG2-*rhlR* KO plasmid and △*rhlR*$_{29\text{-}601bp}$
rhlR-up-F	CACAGCAAAAAGCCTCCGTC	
rhlR-down-F	TCGCCATCATCCTGAGCA	
rhlR-down-R	ATGTAGCGGGTTTGCGGAT	
pilA(PAO1)-upF	GTTGCCACAACCATCGCATC	For constructing the pEXG2-*pilA* (PAO1) K.O. plasmid and △*pilA*$_{54\text{-}417bp}$
pilA(PAO1)-upR	CCGATGTTCACTCCGAAAGGT	
pilA(PAO1)-downF	GATCGCAACCACGATCATCA	
pilA(PAO1)-downR	GGGTCGGAGATGCCTACAAA	

LITERATURE CITED

Chugani, S., B. S. Kim, S. Phattarasukol, M. J. Brittnacher, S. H. Choi, C. S. Harwood, and E. P. Greenberg, 2012, Strain-dependent diversity in the *Pseudomonas aeruginosa* quorum-sensing regulon: Proc Natl Acad Sci U S A, v. 109, p. E2823-31.

Figurski, D. H., and D. R. Helinski, 1979, Replication of an origin-containing derivative of plasmid RK2 dependent on a plasmid function provided in trans: Proc Natl Acad Sci U S A, v. 76, p. 1648-52.

Mendoza, S. D., E. S. Nieweglowska, S. Govindarajan, L. M. Leon, J. D. Berry, A. Tiwari, V. Chaikeeratisak, J. Pogliano, D. A. Agard, and J. Bondy-Denomy, 2020, A bacteriophage nucleus-like compartment shields DNA from CRISPR nucleases: Nature, v. 577, p. 244-248.

Tatusova, T., M. DiCuccio, A. Badretdin, V. Chetvernin, E. P. Nawrocki, L. Zaslavsky, A. Lomsadze, K. D. Pruitt, M. Borodovsky, and J. Ostell, 2016, NCBI prokaryotic genome annotation pipeline: Nucleic Acids Res, v. 44, p. 6614-24.

Varadi, M., D. Bertoni, P. Magana, U. Paramval, I. Pidruchna, M. Radhakrishnan, M. Tsenkov, S. Nair, M. Mirdita, J. Yeo, O. Kovalevskiy, K. Tunyasuvunakool, A. Laydon, A. Žídek, H. Tomlinson, D. Hariharan, J. Abrahamson, T. Green, J. Jumper, E. Birney, M. Steinegger, D. Hassabis, and S. Velankar, 2024, AlphaFold Protein Structure Database in 2024: providing structure coverage for over 214 million protein sequences: Nucleic Acids Res, v. 52, p. D368-D375.

Wang, M., A. L. Schaefer, A. A. Dandekar, and E. P. Greenberg, 2015, Quorum sensing and policing of *Pseudomonas aeruginosa* social cheaters: Proc Natl Acad Sci U S A, v. 112, p. 2187-91.

Wishart, D. S., S. Han, S. Saha, E. Oler, H. Peters, J. R. Grant, P. Stothard, and V. Gautam, 2023, PHASTEST: faster than PHASTER, better than PHAST: Nucleic Acids Res, v. 51, p. W443-W450.

Zimmermann, L., A. Stephens, S. Z. Nam, D. Rau, J. Kübler, M. Lozajic, F. Gabler, J. Söding, A. N. Lupas, and V. Alva, 2018, A completely reimplemented MPI bioinformatics toolkit with a new HHpred server at its core: J Mol Biol, v. 430, p. 2237-2243.
